# Diagnostic characteristics, treatment patterns, and clinical outcomes for patients with advanced/metastatic medullary thyroid cancer

**DOI:** 10.1186/s13044-021-00119-9

**Published:** 2022-02-12

**Authors:** Rohan Parikh, Lisa M. Hess, Elizabeth Esterberg, Naleen Raj Bhandari, James A. Kaye

**Affiliations:** 1 RTI Health Solutions, 3040 East Cornwallis Road, Research Triangle Park, NC 27709 USA; 2 Eli Lilly and Company, Lilly Corporate Center, Indianapolis, IN 46285 USA; 3 RTI Health Solutions, 307 Waverley Oaks Road, Waltham, MA 02452 USA

**Keywords:** *RET*, Medical record, Chart review, Retrospective, Observational, Real-world, United States survival

## Abstract

**Background:**

Medullary thyroid cancer (MTC) accounts for approximately 1.6% of new cases of thyroid cancer. The objective of this study was to describe patient characteristics, biomarker testing, treatment patterns, and clinical outcomes among patients with advanced/metastatic MTC in a real-world setting in the United States and to identify potential gaps in the care of these patients.

**Methods:**

Selected oncologists retrospectively reviewed medical records of patients aged ≥ 12 years diagnosed with advanced MTC. Patients must have initiated ≥ 1 line of systemic treatment for advanced/metastatic MTC between January 2013–December 2018 to be eligible. Patient characteristics, biomarker testing, and treatment patterns were summarized descriptively; progression-free survival (PFS) and overall survival (OS) were estimated using the Kaplan–Meier method.

**Results:**

The 203 patients included in this study had a mean (SD) age of 52.2 (10.4) years; mean (SD) duration of follow-up from start of first-line treatment was 24.5 (16.0) months. Most patients (82.8%) were initially diagnosed with stage IVA, IVB, or IVC disease. Among all patients, 121 (59.6%) had testing for *RET* mutations, of whom 37.2% had *RET*-mutant MTC. The *RET*-mutation type was reported for 28 patients; the most common mutations reported were M918T (64.3%) and C634R (32.1%). Of the 203 patients, 75.9% received only one line of systemic treatment for advanced disease, and 36% were still undergoing first-line therapy at the time of data extraction. Cabozantinib (30.0%), vandetanib (30.0%), sorafenib (17.2%), and lenvatinib (4.9%) were the most common first-line treatments. Among 49 patients who received second-line treatment, most received cabozantinib (22.4%), vandetanib (20.4%), lenvatinib (12.2%), or sunitinib (12.2%). Median PFS (95% confidence interval [CI]) from start of first- and second-line treatments was 26.6 months (20.8–60.8) and 15.3 months (6.6-not estimable [NE]), respectively. Median OS from initiation of first- and second-line treatment was 63.8 months (46.3-NE) and 22.4 months (12.4-NE), respectively.

**Conclusions:**

For the treatment of advanced/metastatic MTC, no specific preference of sequencing systemic agents was observed in the first- and second-line settings. Considering the recent approval of selective *RET* inhibitors for patients with *RET*-mutant MTC, future research should investigate how treatment patterns evolve for these patients.

**Supplementary Information:**

The online version contains supplementary material available at 10.1186/s13044-021-00119-9.

## Background

Medullary thyroid cancer (MTC) is a rare cancer evolving from neural crest–derived calcitonin-producing parafollicular C cells [1]. MTC accounts for 1.6% of all histologically confirmed incident thyroid tumors (1,562/95,669 cases) in the United States (US) [2]. A study of the US Surveillance, Epidemiology, and End Results (SEER) Program for cases diagnosed in 1994–2013 found that MTC accounted for 8.0% of all thyroid cancer–related deaths and 9.1% of age-adjusted thyroid cancer–related mortality during this period^1^ [3]. MTC can be either sporadic or hereditary, the latter occurring either with other endocrine neoplasms (multiple endocrine neoplasia [MEN] types 2A and 2B) or alone (familial MTC). Most MTC cases are characterized by a mutation of the rearranged during transfection (*RET)* proto-oncogene, which can be either germline or somatic [4, 5]. For example, an estimated 65%-90% of sporadic MTCs harbor somatic *RET* mutations [5–7], and autosomal dominant inheritance of an activating *RET* mutation causes hereditary MTC (both MEN2 syndromes and familial MTC) [8].

Approximately half of US patients with MTC are diagnosed with local disease [9]. The primary and curative treatment for most patients diagnosed with early-stage MTC comprises total thyroidectomy and neck dissection [10]. To treat patients with symptomatic advanced, progressive, or recurrent MTC, systemic therapies such as vandetanib, cabozantinib, and, for patients with *RET*-mutant MTC, selpercatinib and pralsetinib are approved by the US Food and Drug Administration and included in national treatment guidelines [10]. With the availability of *RET*-targeted therapies, genetic biomarker testing to identify *RET* alterations should be part of the standard of care for patients with advanced or metastatic MTC.

Evidence suggests that stage at diagnosis, presence and subtype of *RET* mutation, levels of biomarkers such as calcitonin and carcinoembryonic antigen (CEA), and type of systemic treatment may affect prognosis in advanced/metastatic MTC [11–15]. However, limited real-world evidence describes such patients in the US, their diagnostic and treatment patterns, and their clinical outcomes. This retrospective observational study evaluated the patterns of biomarker testing, treatments, and clinical outcomes among patients with advanced or metastatic MTC receiving routine clinical care.

## Methods

### Study design overview

An observational retrospective, medical record review of patients who had a confirmed diagnosis of advanced/metastatic MTC was conducted. Participating oncologists (medical/clinical oncologists or hematologist/oncologists) who had treated ≥ 1 patient with advanced MTC in the year before data abstraction, practiced for ≥ 3 years after completion of formal training or board certification, and were the main decision-maker regarding treatment for their patients with advanced MTC abstracted demographic and clinical data into a customized, web-based case-report form. Oncologists were asked to select a quasi-random sample of their patients by abstracting medical records of those whose last names began with a randomly generated letter. Data abstraction occurred in April–May 2020. Data were then compiled into an analytic data set of deidentified patient-level data. RTI International’s institutional review board (IRB) reviewed the study protocol and deemed the research, which was not considered human subjects research in accordance with the US Code of Federal Regulations (CFR) Sect. 45 CFR 46, to be exempt from full IRB review.

### Study population

Eligible patients had a diagnosis of histologically and/or cytologically confirmed MTC or were initially diagnosed with or had progressed to having locally advanced or metastatic MTC (collectively referred to as “advanced MTC” hereafter). Patients were required to have initiated ≥ 1 line of systemic anticancer treatment (single agent or combination) as their first therapy for advanced MTC (i.e., the eligible systemic therapy) between January 1, 2013, and December 31, 2018, be aged ≥ 12 years at that time, and have a complete medical record covering all treatments after advanced MTC diagnosis. Decisions to initiate therapy were made by the treating physicians. The date of initiation of first-line therapy for advanced MTC was defined as the index date. Patients could be living or deceased at the time of record abstraction. Excluded patients had other malignant neoplasms before the index date (except *MEN2*-associated pheochromocytoma that had been resected or was documented to be stable; nonmelanoma skin cancer; in situ cervical cancer; or other cancer from which the patient had been disease free for ≥ 5 years on the index date) or had participated in a clinical trial of an interventional drug as a first-line systemic treatment for advanced MTC.

Due to the retrospective, descriptive nature of this study, the targeted sample was not based on formal statistical considerations. Based on a feasibility assessment, a sample of approximately 200 patients across the US was planned.

### Study measures

In addition to patient characteristics, baseline information extracted from medical records included tumor stage at initial diagnosis, testing for potential germline or somatic mutations of special interest, and serum CEA and calcitonin levels. Systemic therapies received before and after advanced MTC diagnosis were recorded overall and by line of therapy. The sequence of regimens received for first- and second-line treatments was derived from the individual drug information for each line of therapy after advanced MTC diagnosis. Objective response (complete or partial response) to first- and second-line treatment was reported by each patient’s oncologist. Criteria used by the treating clinician to assess response could include physical examination, performance status, nongenomic biomarker levels (calcitonin or CEA), imaging, or objective criteria (e.g., RECIST guidelines). Progression-free survival (PFS) and overall survival (OS) were estimated from the start of first- and second-line therapies. Clinician-defined disease progression, initiation of subsequent line of treatment, and death were considered progression events and were used to estimate PFS. Patient and tumor characteristics, treatments, and clinical outcomes were summarized for the overall study population and for the subgroup of patients with *RET*-mutant MTC.

### Statistical analyses

All analyses were descriptive and were conducted using SAS (version 9.4, SAS Institute Inc., Cary, North Carolina). Time-to-event outcomes (OS and PFS from initiation of first-line and second-line therapies, respectively) were described using the Kaplan–Meier method. Subgroup analyses were conducted for patients with germline or somatic *RET* mutations. CEA and calcitonin levels were evaluated at advanced MTC diagnosis and during first- and second-line treatment. A post hoc mixed-model repeated-measures analysis accounting for within-patient correlation was conducted to evaluate improvement or decline in calcitonin levels and CEA levels during first-line therapy [16]. Time to decline of ≥ 50% from the calcitonin level and CEA level at initiation of the treatment line (± 28 days) were each estimated from the mixed-model repeated-measures analysis.

## Results

Seventy-five physicians (40 medical/clinical oncologists and 35 hematologist/oncologists) abstracted data from electronic medical records of a total of 203 patients with advanced MTC (per physician: mean, 2.8 patients [standard deviation [SD], 2.0; range, 1–6). The 75 participating physicians represented all geographic regions (Northeast, *n* = 19 [25.3%]; Midwest, *n* = 12 [16.0%]; South, *n* = 23 [30.7%]; and West, *n* = 21 [28.0%]) (Table 1). Most physicians (*n* = 59 [78.7%]) practiced in a cancer center or tertiary referral center (*n* = 34 [45.3%]) or a private hospital or clinic (*n* = 25 [33.0%]); 13 (17.3%) practiced in an academic or teaching hospital, and 3 (4.0%) practiced in a nonteaching hospital setting. The mean (SD) number of years in practice, managing treatment of oncology patients since fully qualified, was 14.7 (5.7) years.

**Table 1:** Characteristics of Participating Physicians

Total physician sample (N)	75	100.0%
Number of patients treated with advanced MTC in the last 12 months		
Mean (SD)	26.0 (20.9)	
Median (IQR)	20.0 (8.0–45.0)	
Primary medical specialty, (n, %)		
Medical or clinical oncology (oncologist)	40	53.3%
Hematology-oncology	35	46.7%
Primary practice setting, (n, %)		
Cancer center/tertiary referral treatment center	34	45.3%
Academic/teaching hospital	13	17.3%
Other nonteaching hospital	3	4.0%
Private hospital or clinic	25	33.3%
Number of years in practice managing treatment of oncology patients since fully qualified		
Mean (SD)	14.7 (5.7)	
Median (IQR)	15.0 (10.0–18.0)	
Geographic region of practice, (n, %)		
Northeast	19	25.3%
Midwest	12	16.0%
South	23	30.7%
West	21	28.0%
Number of patients for whom data was provided for this study		
Mean (SD)	2.8 (2.0)	
Median (IQR)	2.0 (1.0-4.0)	
Min, Max	1.0	6.0

*MTC* medullary thyroid cancer, *SD* standard deviation

### Overall cohort of patients with advanced medullary thyroid cancer

#### Patient and clinical characteristics

At advanced MTC diagnosis, the mean (SD) age of patients included in this study was 52.2 (10.4) years; 58.6% of patients (*n* = 119) were female, and 66.0% (*n* = 134) were white (Table 2). Mean (SD) duration of follow-up was 24.5 (16.0) years. Most patients (*n* = 168; 82.8%) had stage IV MTC (including IVA, IVB, and IVC) at initial diagnosis. Among the 141 patients who underwent evaluation of calcitonin level at advanced MTC diagnosis, 117 (83.0%) had a known calcitonin level (mean [SD], 150.1 [138.9] pg/mL). Among the 108 patients who underwent CEA testing at advanced MTC diagnosis, 84 (77.8%) had a known CEA level (mean [SD], 30.0 [30.4] ng/mL). Among the 173 patients whose performance status at advanced MTC diagnosis was known, 142 (82.1%) had a performance status of 0/1.

**Table 2:** Characteristics of Patients With Advanced Medullary Thyroid Cancer

	**All Patients,** **n (%)**		**Patients With *RET***-Mutant MTC, **n (%)**	
Number of patients, N	203	100.0%	45	100.0%
Age at advanced diagnosis of MTC	203	100.0%	45	100.0%
Mean (SD)	52.2 (10.4)		46.6 (9.7)	
Median (IQR)	53 (44–59)		46 (39–54)	
Sex				
Female	119	58.6%	26	57.8%
Male	84	41.4%	19	42.2%
Race ^a^				
White	134	66.0%	24	53.3%
Black/African American	45	22.2%	10	22.2%
Asian, Native Hawaiian or other Pacific Islander	18	8.9%	6	13.3%
Other ^b^	1	0.5%	1	2.2%
Unknown or not reported	6	3.0%	4	8.9%
Ethnic origin				
Hispanic or Latina/Latino	25	12.3%	5	11.1%
Not Hispanic or Latina/Latino	163	80.3%	33	73.3%
Unknown or not reported	15	7.4%	7	15.6%
Total duration of follow-up, months ^c^	203	100.0%	45	100.0%
Mean (SD)	24.5 (16.0)		28.8 (18.0)	
Median (IQR)	21.1 (15.0–28.5)		25.5 (17.9–34.5)	
Clinical stage at initial diagnosis				
Stage II	10	4.9%	0	0.0%
Stage III	20	9.9%	6	13.3%
Stage IVA	58	28.6%	12	26.7%
Stage IVB	28	13.8%	4	8.9%
Stage IVC	82	40.4%	22	48.9%
Unknown or not reported	5	2.5%	1	2.2%
Time from initial diagnosis to advanced diagnosis date (months), among patients who were initially diagnosed with stage I, II, III (n, %)	30	14.8%	6	13.3%
Mean (SD)	26.3 (33.0)		37.5 (64.0)	
Median (IQR)	14.1 (8.2–28.8)		12.6 (10.1–17)	
Site(s) of local extension or metastasis at advanced MTC diagnosis ^a^	203	100%	25	100%
Distant lymph nodes	111	54.7%	20	44.4%
Bone	69	34.0%	18	40.0%
Brain	14	6.9%	6	13.3%
Liver	56	27.6%	19	42.2%
Local structures (e.g., muscles, larynx, trachea, esophagus, or large vessels)	58	28.6%	12	26.7%
Lung/pleura	81	39.9%	15	33.3%
Performance status at advanced MTC diagnosis (± 30 days) ^d^	173	85.2%	42	93.3%
0 or 1	142	82.1%	36	85.7%
2–4	31	18.0%	6	14.3%
Calcitonin level evaluated at advanced MTC diagnosis (± 30 days) (pg/mL) (n, %) ^e^	141	69.5%	31	68.9%
Calcitonin level known (n, %)	117	83.0%	23	74.2%
Mean (SD)	150.1 (138.9)		139.2 (138.6)	
Median (IQR)	110.0 (22.0–210.0)		100.0 (18.0–200.0)	
Calcitonin evaluated but level unknown or not reported (n, %)	24	17.0%	8	25.8%
CEA test performed at advanced MTC diagnosis (± 30 days) (ng/mL) (n, %) ^f^	108	53.2%	26	57.8%
CEA level known (n, %)	84	77.8%	14	53.8%
Mean (SD)	30.0 (30.4)		27.5 (28.2)	
Median (IQR)	18.3 (3.7–50.0)		19.3 (6.8–30.0)	
CEA tested but level unknown or not reported (n, %)	24	22.2%	12	46.2%

*CEA* carcinoembryonic antigen, *ECOG* Eastern Cooperative Oncology Group, *IQR* interquartile ratio, *MTC* medullary thyroid cancer, *RET* rearranged during transfection, *SD* standard deviation

^a^Multiple responses allowed; rows will add up to greater than 100%

^b^Reported as mixed race

^c^Length of follow-up is the duration of time between the date of initiation of first-line systemic therapy and death or end of patient record

^d^Karnofsky score were converted to the ECOG scale for 29 patients (14.3%) [17]

^e^Calcitonin normal range: < 10 pg/mL

^f^CEA test normal range: < 2.5 ng/mL

Most patients (*n* = 121; 59.6%) underwent biomarker testing for *RET* mutations (Table 3). Of the 45 (37.2%) patients with known *RET-*mutant MTC, 25 (55.6%) had *RET* mutations first identified before advanced MTC diagnosis, and 20 (44.4%) had them first identified after advanced MTC diagnosis. Among patients with *RET* mutations, 18 (40%) had *M918T* mutation, 9 (20%) had *C634R* mutation, 1 (2%) had *C634G* mutation, and the specific type of *RET* mutation was not known/documented for 17 (38%) patients. Most patients had no hereditary clinical syndromes documented (169; 83.3%); 15 patients (7.4%) had a diagnosis of familial MTC, 8 (3.9%) had MEN2A syndrome, and 5 (2.5%) had MEN2B. The data collected did not distinguish between somatic and germline biomarker testing.

**Table 3:** *RET*-Mutation Testing and Diagnosis of Hereditary Medullary Thyroid Cancer Syndromes

	**N**	**%**
Total patient sample (N)	203	100.0%
Tested for germline or somatic *RET* mutation at any time^a^ (n, %)		
Yes	121	59.6%
No/unknown	82	40.4%
Biomarker testing at or after initial diagnosis of MTC		
Patients tested for potential germline and/or somatic mutations at or after initial diagnosis of MTC (n, %)	95	46.8%
Patients with *RET-*mutant MTC (n, %)	45	37.2%
RET mutation identified before initial MTC diagnosis	25	55.6%
RET mutation identified after initial MTC diagnosis	20	44.4%
Type of *RET* mutation among patients with *RET-*mutant MTC (n, %)	45	37.2%
M918T	18	40.0%
C634R	9	20.0%
C634G	1	2.2%
Unknown	17	37.8%
Diagnosis of hereditary MTC syndromes at any time		
MEN2A	8	3.9%
MEN2B	5	2.5%
Familial MTC	15	7.4%
No hereditary MTC syndrome diagnosed	169	83.3%
Unknown/not reported	6	3.0%

*MEN2A* multiple endocrine neoplasia type 2A, *MEN2B* MEN type 2B, *MTC* medullary thyroid cancer; *RET* rearranged during transfection

^a^The data collection form did not distinguish between germline and somatic testing

#### Treatment patterns

The mean time from advanced diagnosis to initiation of first-line therapy was 1.9 (SD = 6.0) months (Table 4). Most patients (*n* = 154; 75.9%) received only one line of systemic anticancer therapy during the available follow-up time; 49 (24.1%) received second-line therapy, and 4 (2.0%) received third-line therapy. Overall, 73 patients (36%) were receiving ongoing first-line treatment at data abstraction. The mean (SD) number of lines of therapy received was 1.3 (0.5); mean (SD) total duration of systemic therapy was 12.0 (11.9) months. The most common first-line therapies received were cabozantinib (*n* = 61, 30.0%), vandetanib (*n* = 61, 30.0%), sorafenib (*n* = 35, 17.2%), and lenvatinib (*n* = 10, 4.9%); Table S-1 (Additional file 1) presents the most common regimens overall.

**Table 4:** Systemic Therapy Patterns Among Patients With Advanced Medullary Thyroid Cancer

	**All MTC Patients**				**Patients With *RET-*Mutant MTC**			
	**First-line Therapy**		**Second-line Therapy**		**First-line Therapy**		**Second-line Therapy**	
Number of patients initiating treatment (N, %)	203	100.0%	49	100.0%	45	100.0%	13	100.0%
Performance status at start of treatment ^a^, (n, %)	138	68.0%	35	71.4%	30	66.7%	9	69.2%
0 or 1	112	81.2%	20	57.1%	25	83.3%	6	66.6%
2–4	26	18.8%	15	42.9%	5	16.7%	3	33.3%
Time from advanced MTC diagnosis to first line of treatment and time between first and second line of treatment, among patients who discontinued first line of treatment (n, %)	203	100.0%	49	100.0%	45	100.0%	13	100.0%
Mean (SD)	1.9 (6.0)		1.3 (2.8)		1.0 (1.4)		0.8 (0.8)	
Median (IQR)	0.5 (0.3–1.2)		0.5 (0.3–0.7)		0.5 (0.3–1.2)		0.5 (0.3–1.2)	
Total duration of therapy line, months (n)	203		49		45		13	
Kaplan–Meier estimate								
Median (95% CI)	12.5 (9.2–18.2)		7.9 (5.8–11.2)		9.7 (6.6–23.7)		NE (6.1-NE)	
Treatment ongoing (n, %)	73	36.0%	16	32.70%	15	33.3%	9	69.20%
Number of patients who discontinued treatment (n, %)	130	64.0%	33	67.3%	30	66.7%	4	30.8%
Reason for discontinuation (n, %) ^b, c^								
Adverse event	3	2.3%	0	0.0%	1	3.3%	0	0.0%
Patient decision	19	14.6%	5	15.2%	4	13.3%	3	75.0%
Progressive disease	70	53.8%	18	54.5%	15	50.0%	1	25.0%
Completion of planned course of treatment	43	33.1%	9	27.3%	11	36.7%	1	25.0%
Loss to follow-up	3	2.3%	0	0.0%	0	0.0%	0	0.0%
Death	9	6.9%	2	6.1%	2	6.7%	0	0.0%
Other: alternative treatment	1	0.8%	0	0.0%	0	0.0%	0	0.0%
Unknown	2	1.5%	1	3.0%	1	3.3%	1	25.0%
Reasons for not administering additional cancer-directed systemic treatment for advanced MTC, **among patients with no subsequent treatment and patients who were alive** at the time of discontinuing last line of treatment (n, %) ^b^	72	35.5%	27	55.1%	15	33.3%	3	23.1%
Patient decision	21	29.2%	6	22.2%	2	13.3%	1	33.3%
Frail physical status	16	22.2%	1	3.7%	2	13.3%	0	0.0%
Stable disease	23	31.9%	5	18.5%	8	53.3%	1	33.3%

*CI* confidence interval, *ECOG* Eastern Cooperative Oncology Group, *IQR* interquartile range, *MTC* medullary thyroid cancer, *PS* performance status, *Q1* first quartile, *Q3* third quartile, *RET* rearranged during transfection, *SD* standard deviation

^a^Karnofsky score converted to the ECOG scale for 29 patients (14.3%) [17]

^b^Categories are not mutually exclusive. A patient may have had more than one reason/criterion assessed; thus, the column does not sum to 100%

^c^Among patients who discontinued treatment line

**Fig. 1:**
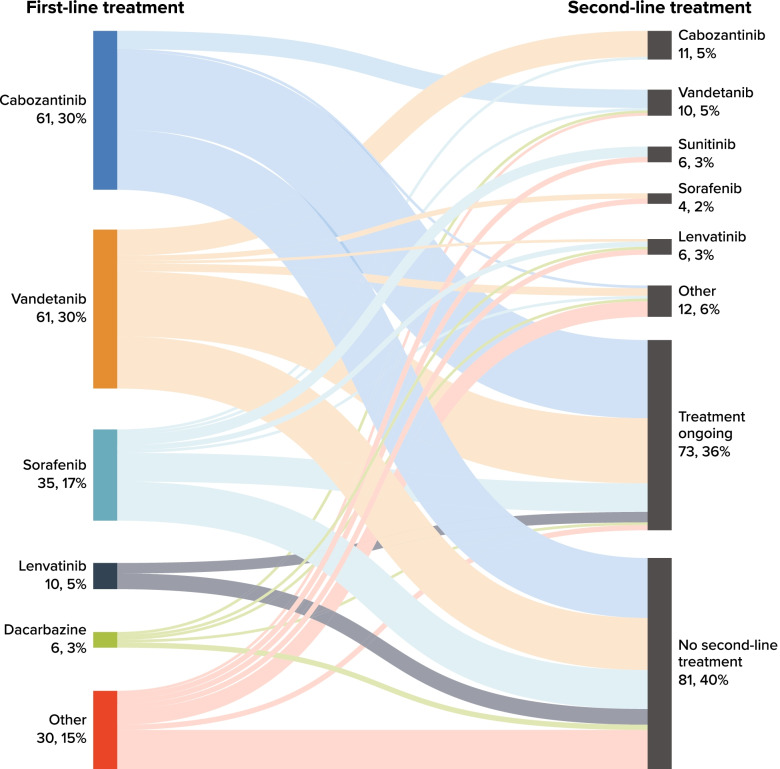
Sankey Chart for Number (%) of Patients Receiving First- and Second-Line Treatment. Note: Only cabozantinib and vandetanib were approved by the US Food and Drug Administration for the treatment of advanced medullary thyroid cancer at the time of the study

Figure 1 presents a Sankey chart of first- and second-line treatment sequences. At the end of study follow-up, 40% of patients had not received a subsequent line of treatment after discontinuing first-line treatment. Similar to first-line treatment, cabozantinib and vandetanib were received by similar proportions of patients in second-line therapy. Of the 61 patients (30%) who received cabozantinib in the first line, 7 (11.5%) received second-line vandetanib; of the 61 patients (30%) who received vandetanib in the first line, 10 (16.4%) received second-line cabozantinib.

During first-line treatment, 129 patients (63.5%) were reported to have an objective response (i.e., complete or partial response); because of ongoing responses at data abstraction, median duration of objective response was not estimable for the 71 patients with known date of objective response (Table 5).

**Table 5:** Objective Response Rate Among Patients With Advanced Medullary Thyroid Cancer

	**All Patients**				**Patients With *RET*-Mutant MTC**			
	**During First-Line Therapy**		**During Second-Line Therapy**		**During First-Line Therapy**		**During Second-Line Therapy**	
Objective response (complete or partial response) (n, %)	129	63.5%	19	38.8%	28	62.2%	8	61.5%
Among those who had objective response and known date of objective response, duration of response (n) ^a^	71		6		12		3	
Mean (SE)	15.4 (1.2)		16.8 (–)		8.4 (0.9)		NE	
Median	NE		NE		NE			
95% CI	10.3	NE	16.8		4.2	NE		
Q1, Q3	6	NE	16.8		6.6	NE		
Censored (n, %)	46	64.8%	5	83.3%	7	58.3%	3	100.0%

*CI* confidence interval, *MTC* medullary thyroid cancer, *NE* not estimable, *Q1* first quartile, *Q3* third quartile, *RET* rearranged during transfection, *SE* standard error

^a^Duration of objective response defined as time from complete or partial response to disease progression, death, or start of next line of treatment. Patients with treatment line ongoing or who discontinued treatment for nonprogression reasons were censored

#### Clinical outcomes

**Fig. 2:**
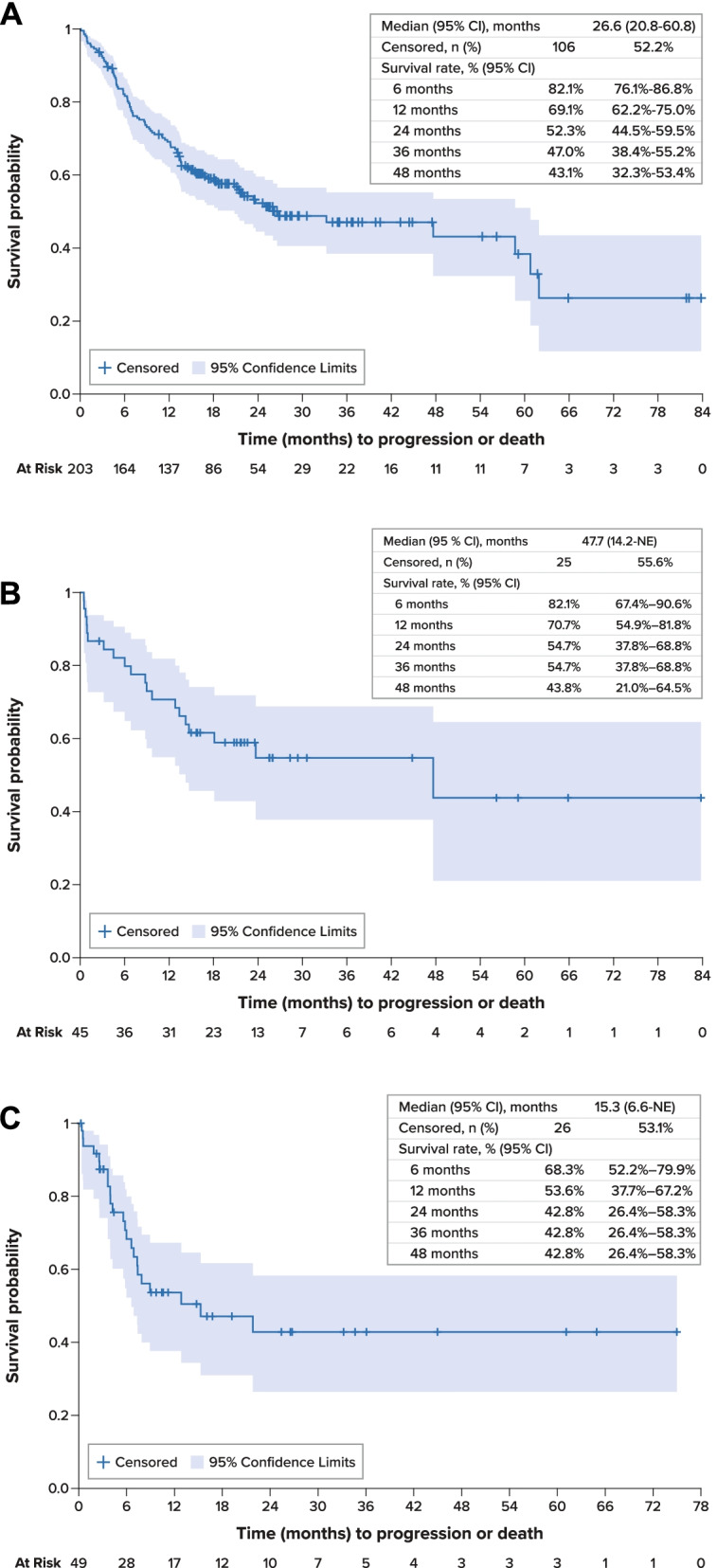
Progression-Free Survival **A**. Overall Population, First-Line Therapy. **B**. *RET*-Mutant Medullary Thyroid Cancer, First-Line Therapy. **C**. Overall Population, Second-Line Therapy. *CI* confidence interval, *MTC* medullary thyroid cancer, *NE* not estimable, *PFS* progression-free survival, *RET* rearranged during transfection, *SE* standard error

**Fig. 3:**
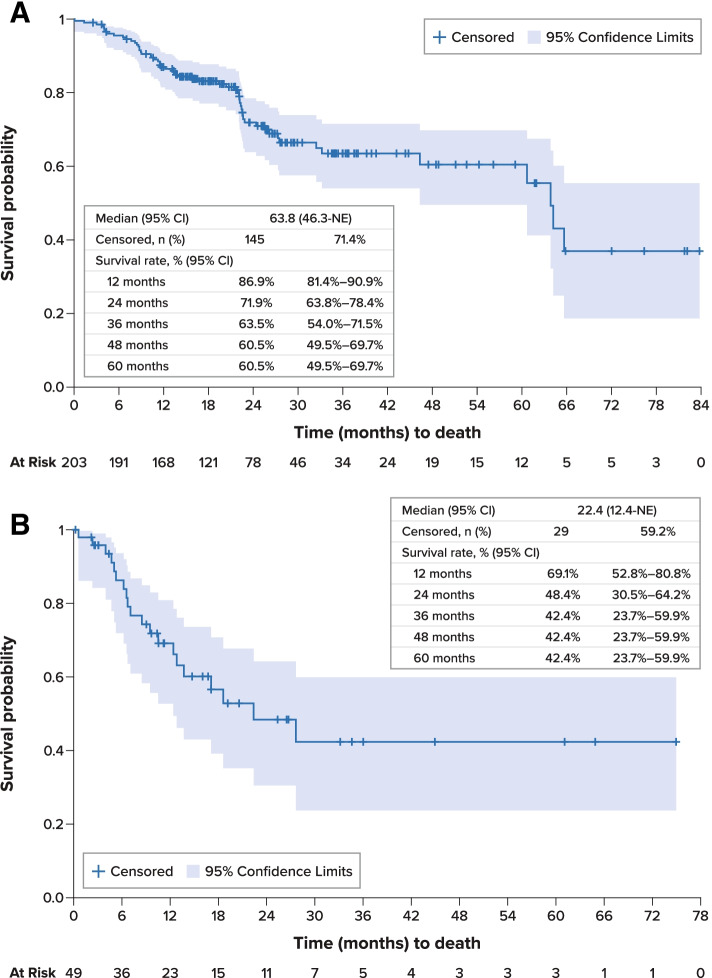
Overall Survival. **A**. From Initiation of First-Line Systemic Treatment. **B**. From Initiation of Second-Line Systemic Treatment. *CI* confidence interval, *NE* not estimable, *SE* standard error

For the overall study cohort, median PFS was 26.6 months (95% confidence interval [CI], 20.8–60.8 months) from initiation of first-line therapy (Fig. 2A). Median OS was 63.8 months (95% CI, 46.3 months–not estimable) from initiation of first-line therapy; survival estimates at 12, 36, and 60 months were 86.9% (95% CI, 81.4%-90.9%), 63.5% (54.0%-71.5%), and 60.5% (49.5%-69.7%), respectively (Fig. 3A). Disease-specific survival at 60 months was 68.3% (95% CI, 58.9%-76.0%).

#### Nongenomic biomarkers

**Fig. 4:**
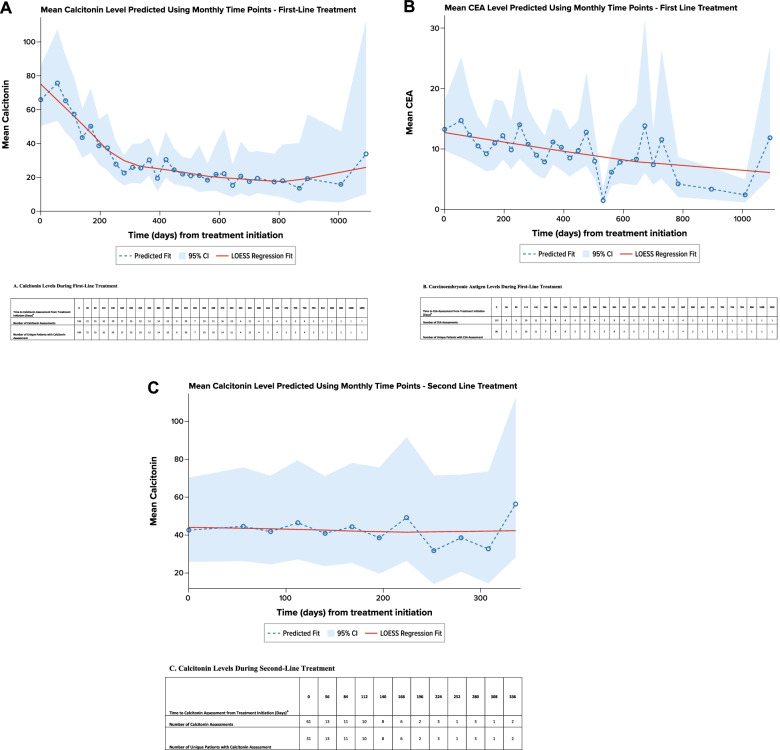
Regression Analysis of Calcitonin and Carcinoembryonic Antigen Levels by Line of Treatment^ a^. **A**. Calcitonin Levels During First-Line Treatment. **B**. Carcinoembryonic Antigen Levels During First-Line Treatment. *CEA* carcinoembryonic antigen. **C**. Calcitonin Levels During Second-Line Treatment. *CEA* carcinoembryonic antigen. Note: A locally weighted polynomial regression, or LOESS, fit was estimated to smooth the data points and highlight the underlying trend [18]. ^a^Predicted using an unadjusted, mixed-model repeated-measures analysis. ^b^Evaluations 28 days before or after initiating treatment line were attributed to baseline (i.e., time 0). Evaluation after 28 days of treatment initiation were grouped in to 28-day intervals and have been assigned to the end of the interval

Calcitonin and CEA levels were evaluated per routine practice (at advanced diagnosis and/or during each treatment line). A total of 109 patients had 159 calcitonin evaluations at the initiation of first-line treatment (± 28 days), and 83 patients had 328 calcitonin evaluations during the first-line treatment; 80 patients had 113 CEA evaluations at the initiation of first-line treatment (± 28 days), and 45 patients had 113 CEA evaluations during the first-line treatment. Regression modeling showed that calcitonin levels generally decreased during first-line treatment, with an estimated time to reach a decrease of ≥ 50% in calcitonin level occurring 8.4 months after treatment initiation (Fig. 4A). Similarly, CEA levels generally decreased during first-line treatment, with an estimated time to reach a decrease of ≥ 50% in CEA level occurring after 17.7 months (Fig. 4B).

### Patients with *RET*-mutant medullary thyroid cancer

The 45 patients with *RET* mutations had a mean (SD) age of 46.6 (9.7) years, and most (*n* = 38; 84.5%) had stage IV disease at advanced MTC diagnosis (Table 2). Among the 42 patients with known performance status at advanced MTC diagnosis, 36 (85.7%) had a performance status of 0/1.

Among patients with *RET* mutations, mean (SD) time from advanced diagnosis to initiation of first-line therapy was 1.0 (1.4) months (Table 4). Most (*n* = 32; 71.1%) received only one line of therapy during the available follow-up time; 12 (26.7%) received second-line therapy, and only 1 (2.2%) received third-line therapy. Fifteen patients (33.3%) were receiving ongoing first-line treatment at data abstraction. The mean (SD) number of lines of therapy received was 1.3 (0.5); mean (SD) total duration of systemic therapy was 9.6 (10.2) months. The most common first-line therapies received were vandetanib (*n* = 18, 40.0%), cabozantinib (*n* = 14, 31.1%), and sorafenib (*n* = 5, 11.1%). During first-line treatment, 28 patients (62.2%) had an objective response; the median duration of objective response was not estimable for the 12 patients with known date of objective response.

Median PFS for patients with *RET*-mutant MTC was 47.7 months (95% CI, 14.2 months–not estimable) from initiation of first-line therapy and was not estimable from initiation of second-line therapy (Fig. 2B). Median OS was not estimable for these patients; survival rates at 12, 36, and 60 months were 95.5% (95% CI, 83.2%-98.9%), 86.8% (70.5%-94.4%), and 86.8% (70.5%-94.4%), respectively.

### Patients undergoing second-line therapy

Forty-nine patients in the overall study cohort (24.1%) received second-line therapy during the available follow-up time. This subgroup had a mean (SD) age of 48.0 (10.5) years at advanced MTC diagnosis. At initiation of second-line therapy, performance status was reported for 35 patients, of whom 20 (57.1%) had a performance status of 0/1, and 15 (42.9%) had a performance status of 2–4. The most frequent second-line treatments were cabozantinib (*n* = 11, 22.4%), vandetanib (*n* = 10, 20.4%), lenvatinib and sunitinib (*n* = 6 [12.2%] for both), and sorafenib (*n* = 4, 8.2%). During second-line treatment, 19 patients (38.8%) had an objective response; the median duration of objective response was not estimable for the 6 patients with known date of objective response. During second-line therapy, regression modeling showed that available calcitonin levels remained generally stable (Fig. 4C). The number of available CEA levels was insufficient for analysis.

From initiation of second-line therapy, median PFS was 15.3 months (95% CI, 6.6 months–not estimable) (Fig. 2C). Median OS was 22.4 months (95% CI, 12.4 months–not estimable), and survival estimates at 12, 36, and 60 months from the initiation of second-line therapy were 69.1% (95% CI, 52.8%-80.8%), 42.4% (23.7%-59.9%), and 42.4% (23.7%-59.9%), respectively (Fig. 3B).

## Discussion

This study retrospectively evaluated clinical characteristics, biomarkers, treatment patterns, and survival outcomes among US patients with advanced MTC managed in real-world clinical settings. Of the patients evaluated, 37.2% had *RET*-mutant MTC. Cabozantinib, vandetanib, sorafenib, and lenvatinib were the most common first-line treatments. Among 49 patients who received second-line treatment, most received cabozantinib, vandetanib, lenvatinib, or sunitinib. For the overall population, median PFS from start of first- and second-line treatments was 26.6 months and 15.3 months; median OS from initiation of first- and second-line treatments was 63.8 months and 22.4 months, respectively.

While real-world studies in MTC have been limited, particularly those evaluating treatment patterns, several recent studies have explored patient and tumor characteristics and treatment outcomes. Randle et al. [9] conducted a population-based study evaluating survival among patients with MTC (of all stages) using 2003–2012 data from the US SEER registry. Overall 5-year disease-specific survival was estimated to be 51% among patients with metastatic MTC [9]. A higher 5-year disease-specific survival rate, 68.3%, was observed for the overall population in the current study. This difference may be attributable to differences in the study time frames, potentially differing prognostic factors, and the introduction of new regimens since Randle and colleagues’ analysis [9].

In addition, a registry study conducted in a routine care setting in Germany enrolled 48 patients with advanced MTC who were treated with the tyrosine kinase inhibitors (TKIs) vandetanib and/or cabozantinib [14]. This population had a median age at diagnosis of metastatic MTC of 50 years and predominantly had sporadic MTC (75% of patients) 13% had hereditary MTC, and germline *RET*-mutation status was not known for 13% of patients. Most patients (96%) had distant metastases. Twelve-month survival estimates were 86% for those receiving vandetanib and 70% for those receiving cabozantinib [14]. The 12-month survival rate of 87.5% observed in the current study is consistent with this prior research, and median duration of treatment was similar in the two studies (25 months in Koehler et al. vs. 21.1 months in the current study). In addition, TKIs were the most commonly administered treatments in the current study: approximately 60% of patients received either cabozantinib or vandetanib in the first line. The TKIs sorafenib and lenvatinib, as well as the cytotoxic drug dacarbazine, were also commonly used in the first line, despite not being approved by the US Food and Drug Administration for the treatment of MTC. Presumably, treating physicians’ off-label use of these therapies was driven by evidence of clinical benefit with sorafenib [19–22], lenvatinib [23], and dacarbazine [24, 25], as well as recommendations in clinical guidelines that small-molecule kinase inhibitors may be used when preferred systemic therapies are not available or appropriate [10].

*RET*-mutation positive MTC has been associated with worse clinical outcomes relative to MTC tumors that do not harbor *RET* mutations [5]. In clinical practice, the proportion of patients undergoing testing for *RET* mutations varies [26], and in the current study, 40% of patients were not known to have undergone testing for germline and/or somatic *RET* mutation. Among the overall sample, 22% of patients were known to have *RET*-mutation positive MTC; these patients had an average age of 46.6 years. Vandetanib monotherapy was the most common first-line regimen for patients with *RET*-mutation positive MTC, followed by cabozantinib monotherapy. The PFS rate at 36 months was 55% after initiation of first-line therapy for this subgroup. To date, studies evaluating real-world treatment patterns for patients with *RET*-mutation positive MTC have been limited, and future research should explore how treatment patterns and outcomes for patients with *RET*-mutant MTC evolve with the availability of the *RET*-targeted therapies selpercatinib and pralsetinib.

Previous studies have found an association between elevated calcitonin and CEA levels, more rapid disease progression, and worse survival outcomes [13, 15]. A mixed-model repeated-measures regression analysis demonstrated that calcitonin levels decreased during first-line treatment initially; appeared to trend upward toward the end of first-line treatment, probably related to disease progression; and remained generally stable during second-line treatment. Because the objective response rate during second-line therapy (among the 49 patients who received it during this study) was lower (39%) than that among all patients during first-line therapy (64%), suboptimal response may potentially explain the pattern of calcitonin levels during second-line therapy, emphasizing the need for more effective treatments.

Several limitations of this study should be considered. Patients selected for study inclusion represent a convenience sample of medical records obtained from physicians willing to participate in the study and may be biased toward patients who were alive at data abstraction; study findings may not be generalizable to the overall population of US patients with advanced MTC. To help mitigate potential biases, physicians were recruited from a variety of regions and practice types and were instructed to select patients who were either alive or dead by a quasi-random procedure. Data available for study were limited to those recorded in medical records. Although internal data consistency was improved by data checks and use of a customized data-collection form, the entered data were not validated against patients’ medical records by independent review. The information collected on genomic biomarker testing did not distinguish between germline and somatic mutations, and the frequency of such testing may have increased in current practice. Finally, a considerable proportion of patients were undergoing treatment at the time of data abstraction, and 52% of patients were censored for PFS estimates; therefore, PFS estimates are based on immature data, and studies with longer follow-up are warranted in this population.

## Conclusions

More than one-third of patients with advanced or metastatic MTC were not tested for *RET* mutation as recommended by national guidelines. For the treatment of advanced/metastatic MTC, no specific preference of sequencing systemic agents was observed in the first- and second-line settings. The estimated OS was consistent with that observed from SEER data for metastatic MTC. Considering the recent approval of selective *RET* inhibitors for patients with *RET*-mutant MTC, future research should investigate potential changes in these findings, particularly in the second-line setting. Evidence-based recommendation on sequencing of systemic therapies may benefit patients with advanced MTC.

## Supplementary Information

**Additional file 1:** **Table S1.** Most Common First- and Second-Line Systemic Therapies Among Patients With Advanced Medullary Thyroid Cancer.

## Data Availability

Not applicable.

## Abbreviations

CEA: Cost-effectiveness analysis
CI: Confidence interval
IRB: Institutional review board
MTC: Medullary thyroid cancer
NCCN: National Comprehensive Cancer Network
OS: Overall survival
PFS: Progression-free survival
RET: Rearranged during transfection
SD: Standard deviation
SEER: Surveillance, Epidemiology, and End Results
TKI: Tyrosine kinase inhibitor
US: United States

## Footnotes

1 The reported age-adjusted annual incidence-based mortality estimates per 100,000 for MTC and for all thyroid cancers were 0.04 (95% confidence interval [CI], 0.03–0.04) and 0.44 (95% CI, 0.42–0.46), respectively, and 0.04/0.44 = 9.1%.
