# Supplementary material for: Diagnostic characteristics, treatment patterns, and clinical outcomes for patients with advanced/metastatic medullary thyroid cancer
Source: Thyroid Res. 2022 Feb 12;15:2. doi: 10.1186/s13044-021-00119-9 (PMC8840546; doi:10.1186/s13044-021-00119-9)
Supplement: Supplementary file 1 — Additional file 1: Table S1. Most Common First- and Second-Line Systemic Therapies Among Patients With Advanced Medullary Thyroid Cancer. [file 13044_2021_119_MOESM1_ESM.docx]

# SUPPLEMENTAL APPENDIX

Table S-1. Most Common First- and Second-Line Systemic Therapies Among Patients With Advanced Medullary Thyroid Cancer

|  | All MTC Patients | | | | Patients With *RET*-mutant MTC | | | |
| --- | --- | --- | --- | --- | --- | --- | --- | --- |
|  | First-Line Therapy | | Second-Line Therapy | | First-Line Therapy | | Second-Line Therapy | |
| Pharmacological agent (given as monotherapy or in combination with other agents)^a^ (n, %) |  |  |  |  |  |  |  |  |
| Cabozantinib | 63 | 31.0% | 12 | 24.5% | 14 | 31.1% | 4 | 30.8% |
| Vandetanib | 62 | 30.5% | 11 | 22.4% | 19 | 42.2% | 2 | 15.4% |
| Dacarbazine | 10 | 4.9% | 1 | 2.0% | 1 | 2.2% | 1 | 7.7% |
| Doxorubicin | 9 | 4.4% | 4 | 8.2% | 2 | 4.4% | 0 | 0.0% |
| 5-fluorouracil | 6 | 3.0% | 2 | 4.1% | 0 | 0.0% | 1 | 7.7% |
| Sorafenib | 35 | 17.2% | 4 | 8.2% | 5 | 11.1% | 2 | 15.4% |
| Sunitinib | 5 | 2.5% | 6 | 12.2% | 0 | 0.0% | 0 | 0.0% |
| Lenvatinib | 11 | 5.4% | 6 | 12.2% | 2 | 4.4% | 2 | 15.4% |
| Pazopanib | 1 | 0.5% | 1 | 2.0% | 1 | 2.2% | 1 | 7.7% |
| Carboplatin | 1 | 0.5% | 0 | 0.0% | 0 | 0.0% | 0 | 0.0% |
| Cisplatin | 8 | 3.9% | 2 | 4.1% | 1 | 2.2% | 0 | 0.0% |
| Dabrafenib | 1 | 0.5% | 0 | 0.0% | 0 | 0.0% | 0 | 0.0% |
| Docetaxel | 4 | 2.0% | 1 | 2.0% | 0 | 0.0% | 0 | 0.0% |
| Entrectinib | 0 | 0.0% | 0 | 0.0% | 0 | 0.0% | 0 | 0.0% |
| Larotrectinib | 0 | 0.0% | 1 | 2.0% | 0 | 0.0% | 0 | 0.0% |
| Paclitaxel | 4 | 2.0% | 2 | 4.1% | 1 | 2.2% | 0 | 0.0% |
| Trametinib | 1 | 0.5% | 0 | 0.0% | 1 | 2.2% | 0 | 0.0% |
| Other | 0 | 0.0% | 0 | 0.0% | 0 | 0.0% | 0 | 0.0% |
| Rationale for prescribing treatment after diagnosis of advanced MTC^a^ (n, %) |  |  |  |  |  |  |  |  |
| In compliance with national guidelines | 137 | 67.5% | 26 | 53.1% | 25 | 55.6% | 8 | 61.5% |
| In compliance with local guidelines | 51 | 25.1% | 5 | 10.2% | 11 | 24.4% | 1 | 7.7% |
| Treatment efficacy | 107 | 52.7% | 30 | 61.2% | 30 | 66.7% | 9 | 69.2% |
| Safety | 58 | 28.6% | 19 | 38.8% | 14 | 31.1% | 7 | 53.8% |
| Patient’s overall health | 36 | 17.7% | 11 | 22.4% | 9 | 20.0% | 3 | 23.1% |
| Disease characteristics | 32 | 15.8% | 15 | 30.6% | 7 | 15.6% | 2 | 15.4% |
| Patient’s request | 9 | 4.4% | 4 | 8.2% | 2 | 4.4% | 4 | 30.8% |
| Convenience of administration | 21 | 10.3% | 2 | 4.1% | 2 | 4.4% | 1 | 7.7% |
| Don’t know | 1 | 0.5% | 0 | 0.0% | 0 | 0.0% | 0 | 0.0% |
| Total duration of therapy line, months (n) | 203 | | 49 | | 45 | | 13 | |
| Kaplan-Meier estimate |  |  |  |  |  |  |  |  |
| Median (95% CI) | 12.5 (9.2-18.2) | | 7.9 (5.8-11.2) | | 9.7 (6.6-23.7) | | NE (6.1-NE) | |
| Treatment ongoing (n, %) | 73 | 36.0% | 16 | 32.70% | 15 | 33.3% | 9 | 69.20% |
| Number of patients who discontinued treatment (n, %) | 130 | 64.0% | 33 | 67.3% | 30 | 66.7% | 4 | 30.8% |
| Adverse event | 3 | 2.3 | 0 | 0.0% | 1 | 3.3% | 0 | 0.0% |
| Patient decision | 19 | 14.6% | 5 | 15.2% | 4 | 13.3% | 3 | 75.0% |
| Progressive disease | 70 | 53.8% | 18 | 54.5% | 15 | 50.0% | 1 | 25.0% |
| Completion of planned course of treatment | 43 | 33.1% | 9 | 27.3% | 11 | 36.7% | 1 | 25.0% |
| Loss to follow-up | 3 | 2.3% | 0 | 0.0% | 0 | 0.0% | 0 | 0.0% |
| Death | 9 | 6.9% | 2 | 6.1% | 2 | 6.7% | 0 | 0.0% |
| Other: alternative treatment | 1 | 0.8% | 0 | 0.0% | 0 | 0.0% | 0 | 0.0% |
| Unknown or not reported | 2 | 1.5% | 1 | 3.0% | 1 | 3.3% | 1 | 25.0% |
| Reasons for not administering additional cancer-directed systemic treatment for advanced MTC, **among patients with no subsequent treatment who were alive** at the time of discontinuing last line of treatment (n, %)^a^ | 72 | 35.5% | 27 | 55.1% | 15 | 33.3% | 3 | 23.1% |
| Patient decision | 21 | 29.2% | 6 | 22.2% | 2 | 13.3% | 1 | 33.3% |
| Frail physical status | 16 | 22.2% | 1 | 3.7% | 2 | 13.3% | 0 | 0.0% |
| Stable disease | 23 | 31.9% | 5 | 18.5% | 8 | 53.3% | 1 | 33.3% |

CI = confidence interval; MTC = medullary thyroid cancer; *RET* = rearranged during transfection.

^a^ Categories are not mutually exclusive. A patient may have had more than one reason/criterion assessed; thus, the column does not sum to 100%.
